# Tendon graft through the coracoid tunnel versus under the coracoid for coracoclavicular/acromioclavicular reconstruction shows no difference in radiographic or patient-reported outcomes

**DOI:** 10.1007/s00402-024-05461-9

**Published:** 2024-07-18

**Authors:** Juha O. Ranne, Terho U. Kainonen, Kari J. Kanto, Janne T. Lehtinen, Pekka T. Niemi, Harry Scheinin

**Affiliations:** 1 Hospital Mehiläinen Neo, 20520 Joukahaisenkatu 6Turku, Finland; 2 Department of Physical Activity and Health, The Paavo Nurmi Centre, The University of Turku, Turku, Finland; 3 Pihlajalinna Hospital, Tampere, Finland; 4 Department of Orthopedics and Traumatology, Tampere University Hospital, Tampere, Finland; 5 Department of Anesthesiology and Intensive Care, University of Turku, Turku, Finland; 6 Division of Perioperative Services, Intensive Care and Pain Medicine, Turku University Hospital, Turku, Finland

**Keywords:** AC dislocation, CC reconstruction, AC reconstruction, Tunnel widening

## Abstract

**Introduction:**

The purpose of this prospective study was to report the outcomes of two different methods in CC and AC reconstruction for the treatment of AC separation using a tendon graft and knot-hiding titanium clavicular implant.

**Materials and methods:**

Twenty-seven patients with Rockwood grade III and V acromioclavicular (AC) separations were randomized into two groups. The primary outcome was whether taking the tendon graft through the coracoid risked a fracture. The following were secondary outcomes: follow-up of clavicular wound healing and Nottingham Clavicle score, Constant score, and Simple Shoulder Test results obtained preoperatively and 24 months postoperatively. The anteroposterior radiographic change between the clavicular and coracoid cortexes and the clavicular tunnel diameter was measured postoperatively and 24 months postoperatively. General patient satisfaction with the outcome (poor, fair, good, or excellent) was assessed 2 years postoperatively.

**Results:**

No coracoid fractures were detected. No issues in clavicular wound healing were detected. The mean Nottingham Clavicle score increased from a preoperative mean of 42.42 ± 13.42 to 95.31 ± 14.20 (*P* < 0.00). The Constant score increased from a preoperative mean of 50.81 ± 17.77 to 96.42 ± 11.51 (*P* < 0.001). The Simple Shoulder Test score increased from a preoperative mean of 7.50 ± 2.45 to 11.77 ± 1.18 (*P* < 0.001). The changes were significant. The coracoclavicular distance increased from 11.88 ± 4.00 to 14.19 ± 4.71 mm (*P* = 0.001), which was significant. The clavicular drill hole diameter increased from 5.5 to a mean of 8.00 ± 0.75 mm. General patient satisfaction was excellent.

**Conclusions:**

There were no significant differences between the two groups. There were no implant related complications in the clavicular wound healing. The results support the notion that good results are achieved by reconstructing both the CC and AC ligaments with a tendon graft.

**Study registration:**

This clinical trial was registered on Clinicaltrials.gov.

## Introduction

An acromioclavicular (AC) dislocation typically occurs when falling on the shoulder. The patients are often young and active individuals. There is general agreement that grade I–II AC dislocations should be treated conservatively, while high-grade dislocations are often treated operatively [1]. In Rockwood grade III dislocations, the AC and CC ligaments are completely torn, and the distal clavicle appears elevated. In grade V dislocations, the surrounding muscle insertions have also been injured, and the distal clavicle is pronouncedly elevated. In grade IV injuries, the distal clavicle is dislocated posteriorly into the trapezius muscle fibers. In rare cases of grade VI dislocations, the distal clavicle is dislocated underneath the coracoid process [2]. The CC ligament complex and the AC joint capsule are the main stabilizers of the distal clavicle. The CC ligaments provide vertical stability to the distal clavicle, whereas AC ligaments provide anteroposterior stability, and considerable forces are exerted on the clavicular ligaments [3–9]. Insufficiency of the distal clavicle may lead to biomechanical problems and scapular dyskinesia [10–12].

Conservative treatment is typically also offered for type III dislocations. However, in some cases, the distal clavicle remains unstable and painful, and operative treatment may be needed even in grade III dislocations. Athletes and younger active patients often tend to receive operative treatment [13, 14]. Numerous techniques have been introduced for the treatment of AC joint dislocations. Creating an operative technique to reconstruct the damaged coracoclavicular ligament system has proven to be challenging. Earlier techniques have included temporary fixations with screws, pins, and plates. Previous arthroscopic techniques included washers, buttons, and interference screws for graft fixation. According to the latest reports using a tendon graft gives the best results in AC separations. [15–17] The complication rates in surgical treatment can be quite high and appear to be related to reconstruction failure, clavicular or coracoid fracture, and/or infections [18–21]. The foreign material may also induce wound irritations and persistent palpable resistances underneath the clavicular wound [22]. The treatment becomes even more difficult if the dislocation is chronic [23]. Three weeks after the trauma, spontaneous healing of the ligament remnants can hardly be expected [24]. Moreover, after 6 weeks, the rupture can be considered chronic [25]. Technical issues, complications and limited longevity of reconstructions have been recurring issues in these operations [18–21].

A critical aspect of achieving reliable coracoclavicular (CC) reconstruction is the utilization of a tendon graft to recreate the torn trapezoid and conoid ligaments, thereby preventing vertical elevation [22–25]. The AC joint capsule also needs to be assessed [7, 26]. Hence, reconstruction of both the CC and AC ligaments with a tendon graft is necessary to attain a sustainable and stable outcome [26–35]. In CC reconstructions, clavicular and subcoracoid implants connected by a strong suture or tape are used to lower the elevated clavicle to the coracoid. There is particular concern about the potential widening of the clavicular and coracoid drill holes, which may pose a risk of fracture or implant sinking [36–39]. When using these described techniques, the graft may be wrapped around the coracoid or taken through a coracoid drill hole, which may increase the fracture risk [22, 38–40].

The purpose of this study was to report the outcomes of CC ligament reconstructions by taking the tendon graft through a 4.5-mm coracoid drill hole or wrapping it around the coracoid.

The hypothesis was that taking the tendon graft through a coracoid drill hole may risk a fracture.

## Methods

This study was reported according to the CONSORT guidelines [41]. This study was approved by the University ethics committee and the institutional research board and was registered on Clinicaltrials.gov.

The original aim, according to the study protocol, was to include 40 patients during a 2-year recruitment period. The recruitment period was from September 1, 2018, to October 19, 2020. However, major difficulties in recruitment were encountered because of the onset of the COVID-19 pandemic. The surgeries were conducted by 4 experienced shoulder surgeons in two hospitals. Twenty-seven patients with Rockwood grade III and V AC separations were randomized and treated surgically using semitendinosus autografts and knot-hiding titanium implants. The inclusion criteria were individuals aged 16 to 70 who were motivated to adhere strictly to postoperative treatment. The interval from trauma to surgery varied from 2 weeks to 3 years. All patients provided written formal consent.

Eleven of the patients had grade V separations, and 7 had grade III separations. Notably, there was one patient who had a revision case (grade V) in the UNDER group and one patient who had a primary lateral clavicle fracture (grade III) in the UNDER group. The techniques and implants used in this trial are routinely used in the two centers where this study was conducted and therefore posed a minimal risk to the patients’ well-being.

Surgery was indicated for Grade III and V patients experiencing pain, distal clavicle instability, and scapular issues The patients were selected in the order they were seeking for operative treatment. Patients were randomly assigned to one of the two groups. Each patient’s allocation was determined by selecting a sealed envelope upon their inclusion in the study. The envelopes consisted of an equal number of slips labeled “Under” (Group UNDER) and “Through” (Group THROUGH), denoting the course of the tendon graft. Finally, the original order of the patients was scrambled.

In the THROUGH group, the tendon graft was passed through a 5.5-mm clavicular drill hole and a 4.5-mm coracoid drill hole [42]. In the UNDER group, the graft was passed through a 5.5-mm clavicular drill hole and then wrapped around the coracoid [43]. Therefore, the clavicular drill hole was similar in both groups. In both groups, the superior AC ligament was also openly reconstructed after arthroscopic CC reconstruction.

The primary outcome measure was whether taking the tendon craft through a 4.5-mm coracoid drill hole would induce a fracture. The secondary outcome measures were clavicular wound healing, changes in shoulder scores, and postoperative radiological changes. Postoperatively, the patients underwent check-ups at 2 weeks, 2 months, and 24 months. During each visit, clavicular wound healing assessed. Preoperatively and 24 months after the surgery, the Nottingham Clavicle Score, Constant Score, and Simple Shoulder Test score were calculated [44–46]. Additionally, an anteroposterior radiograph was obtained at 2 weeks postoperatively and again at the 24-month mark. At the 24-month follow-up, measurements were taken to evaluate the changes in distance between the clavicular and coracoid cortex, as well as the clavicular tunnel diameter. Furthermore, general patient satisfaction with the outcome was assessed using a rating scale of poor, fair, good, and excellent.

A regular power analysis was not conducted because with the resources available in the two-year time frame, the maximal realistic number of patients was 40. Statistical analyses were conducted using Microsoft Excel for Mac 16.71 with the Analysis Tool Pak and Solver add-ins. This software provided descriptive univariate statistics, such as the arithmetic mean and standard deviation, as well as 95% confidence intervals (CIs), t tests, and graphical representations. T tests were performed as two-sample tests with unequal variances for comparison between UNDER and THROUGH population. Paired two sample t-test was used for comparison between 0 and 2 years populations. These tests were used to determine differences between pre- and postoperative groups and to compare the populations of the “THROUGH” (Group 1) and “UNDER” (Group 2) groups. CIs were calculated as the mean ± margin of error (ME), with the ME computed using the confidence.norm function in Excel. Nonlinear calculations for patient satisfaction were performed using RStudio 2022.12.0 + 353 with the binom 1.1–1.1 and boot 1.3–28 libraries.

### Surgical technique

**Fig. 1:**
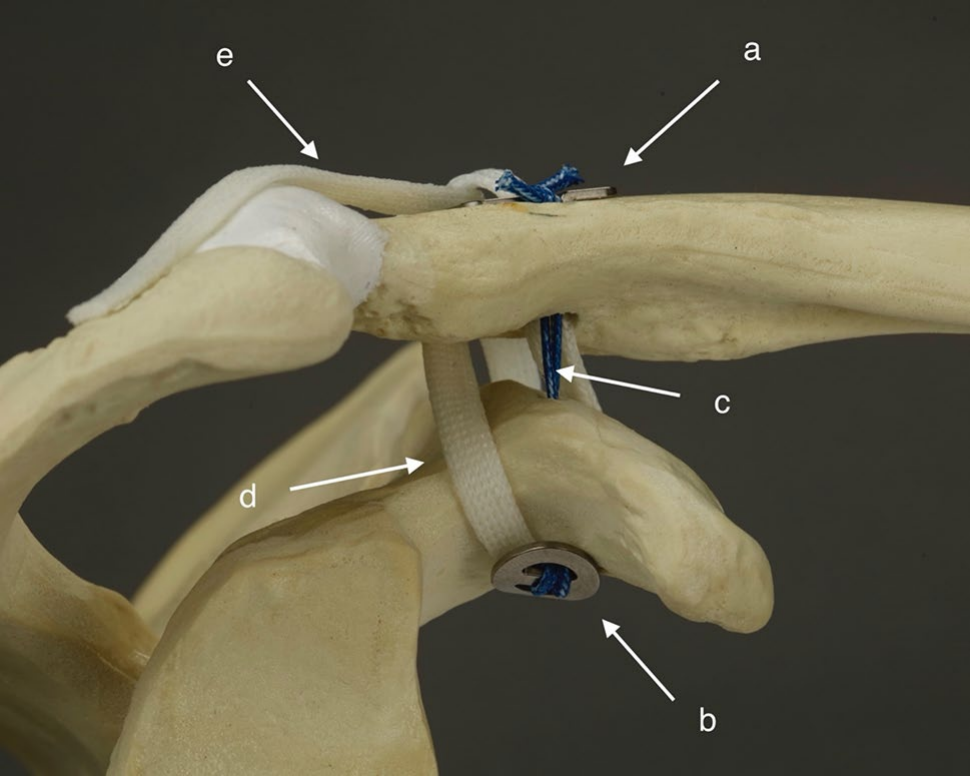
Model of the right shoulder, lateral view. Clavicular clip (**a**), subcoracoid clip (**b**), interconnecting double-folded No. 5 suture (**c**), posterior limb of the tendon graft (**d**) and posterior limb extending over the AC joint (**e**). The figure is of an *UNDER* reconstruction. *THROUGH* reconstruction is otherwise similar, but the tendon graft shares the coracoid drill hole with the interconnecting suture

**Fig. 2:**
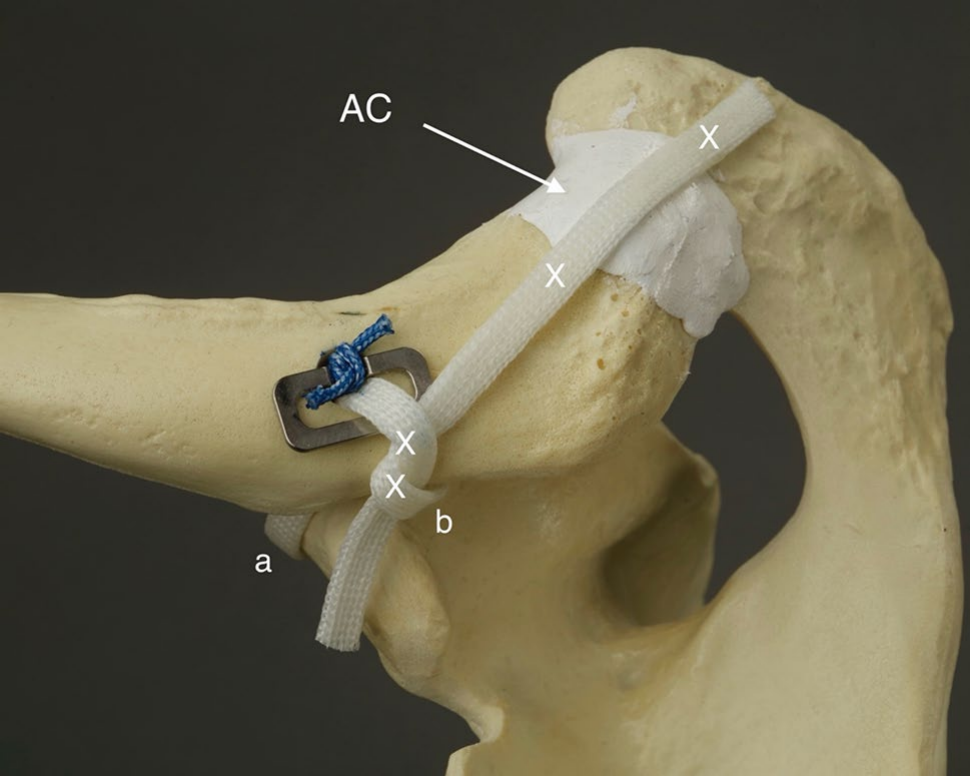
Model of the right shoulder, posterosuperior view. The reconstructed CC ligament. The anterior limb (**a**) and the posterior limb (**b**). The reconstructed AC ligament (AC) and the sites of interrupted No. 2 sutures for fixation and stabilization of the tendon graft (X)

In this study, CC-Clip^®^ titanium implants (CC-Instruments, Baltimore, MD) with double-folded No. 5 interconnecting sutures were used to stabilize the distal clavicle. The clavicular knot-hiding CC-Clip^®^ implants can also be used with a tendon graft. As a knot-hiding device, the system is supposed to reduce clavicular wound issues. The device also allows tendon graft extension over the AC joint to reconstruct the torn superior AC ligament. In this study, a semitendinosus autograft was used (Figs. 1, 2).

Each patient was positioned in the beach chair position, and a standard 30-degree arthroscope was utilized. The surgical technique involved four portals: the posterior, the lateral, the anterolateral, and the clavicular portals. Arthroscopy was initiated by inserting the arthroscope into the joint through the posterior portal. The lateral portal was created by inserting a needle in front of the long-head biceps tendon, targeting the proximal coracoid. This allowed for the exposure of the coracoid neck and subsequent opening of the interval in that area. Once adequate access to the coracoid neck was achieved, the arthroscope was moved to the lateral portal, which served as the primary camera position during the actual reconstruction. The anterolateral portal was established using a needle directed toward the coracoid neck. Debridement was performed and thorough exposure was achieved around the coracoid and clavicle. To expose the superior surface of the clavicle for drilling, a longitudinal incision was made measuring 2.5 cm medially from the acromioclavicular joint. A blunt tissue passageway was created behind the clavicle through the same opening to facilitate subsequent graft passage.

*In the THROUGH group,* a 2.4-mm guide pin was inserted through the clavicle and coracoid using a drill guide under arthroscopic visual control. The clavicular drill hole was centrally located on the clavicle, approximately 2.5 cm proximal to the acromioclavicular joint, under visual control. A 2.4-mm guide pin was used. The pin was positioned centrally and proximally within the bone. A 4.5-mm drill hole was created through the clavicle and coracoid, which was subsequently widened to 5.5 mm for the clavicular drill hole. The passing sutures for the tendon graft and the No. 5 interconnecting suture loop were threaded through the clavicular and coracoid drill holes with the aid of a Nitinol lasso and the Straight Lasso Guide. The tendon graft was first pulled through the clavicular and coracoid drill holes. The distal graft limb was then pulled out dorsally to the clavicle through the clavicular wound. The interconnecting suture loop was then pulled through the clavicular and coracoid drill holes and brought out through the anterolateral portal [41].

*In the UNDER group,* a 2.4-mm guide pin was inserted through the clavicle and coracoid using a drill guide under arthroscopic visual control. The clavicular drill hole was then widened to 5.5 mm. The Curved Lasso Guide was positioned in front of the clavicle and medial to the coracoid while taking the tip of the guide around the coracoid. An additional portal could be opened for the guide in front of the clavicle. The Nitinol wire was passed through the guide, and a suture passer was used to pass the proximal wire end through the clavicular drill hole. The passing suture for the tendon graft was then set into the wire loop and pulled through the clavicular drill hole and guided medially under the coracoid. Subsequently, the tendon graft was pulled through the clavicular drill hole and looped around the coracoid. The distal graft limb was pulled out to the clavicle dorsally through the clavicular wound. The interconnecting suture loop was then passed through both the clavicular and coracoid drill holes, facilitated by a Nitinol lasso and the Straight Lasso Guide, and brought out through the anterolateral portal [42].

The remainder of the surgical procedure was consistent in both groups. The Subcoracoid Clip was fastened to the interconnecting suture loop at the anterolateral portal and pulled into place beneath the coracoid. The ends of the interconnecting suture and the anterior graft limb were taken through the Clavicular Clip eyelet. The dorsal graft limb was left longer for later AC reconstruction.

After completing the arthroscopic CC reconstruction, the clavicular incision was extended over the AC joint. The overstretched AC joint capsule was then dissected along its fibers. To facilitate repositioning, soft tissue attachments and scar tissue surrounding the distal clavicle were released. The distal end of the clavicle was resected using an oscillating saw for the same reason. With the entire reconstruction in place, the clavicle was repositioned and visually assessed for proper reduction. The interconnecting suture and tendon graft of the CC reconstruction were tensioned, and the interconnecting sutures were securely tied in the clavicular clip loop using a knot pusher. The ends of the graft limb were then tensioned, tied to each other, and secured using No. 2 non-resorbable sutures. Finally, the superior AC ligament was reconstructed using the longer dorsal end of the tendon graft. The graft end was sutured on both sides of the AC joint, and the AC capsule was then tightly plicated over it using strong interrupted sutures. The arthroscopic portals were closed using interrupted sutures, while the clavicular wound was closed in layers.

### Postoperative treatment

The patients were discharged on the same day of the surgery and instructed to wear an arm sling for 6 weeks. During this period, they could engage in gentle rotational movements and passive arm lifting within their pain tolerance. After 6 weeks, the sling was no longer used; however, the initiation of gradual rehabilitation was delayed until eight weeks postsurgery to allow for sufficient graft integration to the surroundings. At 3–4 months postsurgery, patients were cleared to resume heavy labor, while overhead activities and contact sports were not permitted until 6 months postsurgery.

## Results

Except for one patient, all participants were male. The mean age (SD) of the patients was 42.3 (12.4) years in the THROUGH group and 43.4 (17.3) years in the UNDER group (Table 1). The exclusion criteria included patients with excessive additional trauma, such as rotator cuff tears requiring repair or significant deviations from the standard operation technique or postoperative treatment. Of the original 27 patients, one was later excluded from this study due to a significant deviation in the operation technique, specifically the absence of AC ligament reconstruction. As a result, 26 patients (ten in the THROUGH group and 16 in the UNDER group) were included in the analysis. Ten of the patients had grade V separations, and 6 had grade III separations.

**Table 1:**
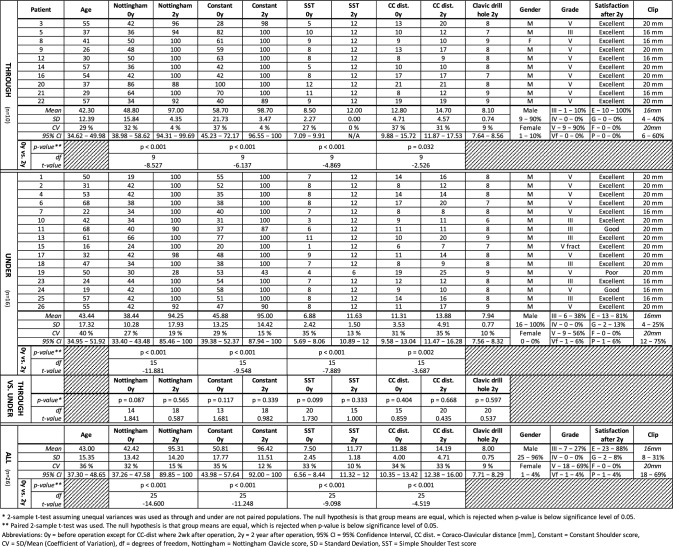
Individual patient data and summary of within group results

No coracoid fractures occurred. No complaints regarding protruding knots on the clavicle or clavicular wound infections were reported. In the UNDER group, a clavicular fracture occurred 8 months after surgery, specifically through the clavicular drill hole, following a new forceful trauma. However, the ligament reconstruction remained unaffected, and the fracture healed with conservative treatment. Additionally, in one patient in the UNDER group, arthroscopic debridement of the AC joint was performed 1 year after the initial operation due to discomfort and cracking in the AC joint, and it healed successfully.

**Fig. 3:**
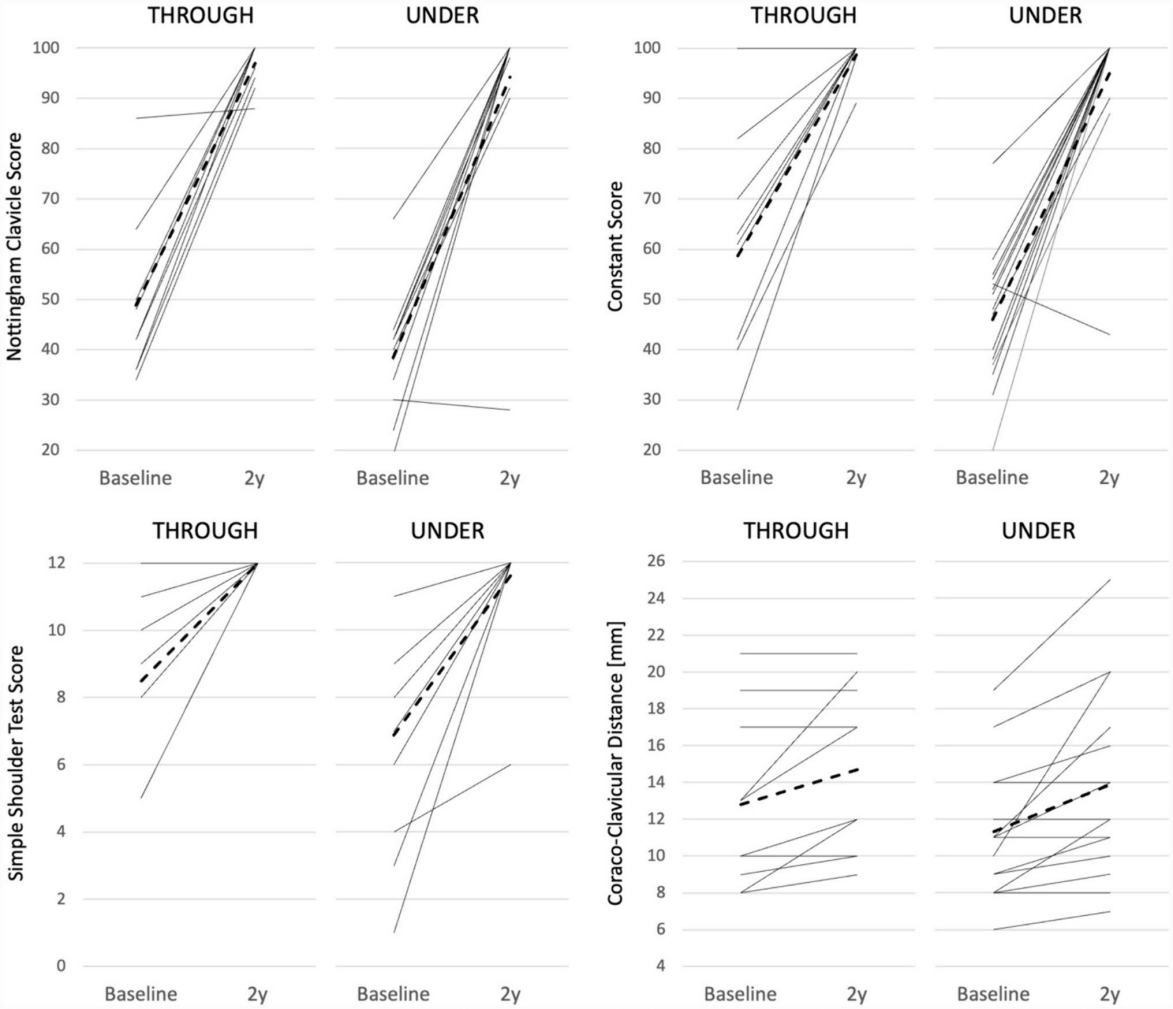
The Nottingham Clavicular Score, The Constant Score, the Simple Shoulder Test Score and coracoclavicular distance at baseline and two years after the operation in the THROUGH and UNDER groups. There were no statistically significant differences in the different efficacy scores or their changes between the groups

There were no statistically significant differences observed in the efficacy scores or their changes between the THROUGH and UNDER groups (Tables 1, 2 and Fig. 3). There was a modest increase in the coracoclavicular distance in both groups The differences between the groups were statistically significant. For descriptive and inferential statistics, see Tables 1 and 2.

**Table 2:** Changes in efficacy scores and summary of between groups results

	2–0 years difference (positive mean = improved score/larger distance after 2 years)			
	Nottingham	Constant	SST	CC dist.
*THROUGH (n = 10)*				
Mean	48.20	40.00	3.50	1.90
SD	17.87	20.61	2.27	2.38
CV	37%	52%	65%	125%
95% CI	37.12–59.28	27.22–52.78	2.09–4.91	0.43–3.37
*UNDER (n = 16)*				
Mean	55.81	49.13	4.75	2.56
SD	18.79	20.58	2.41	2.78
CV	34%	42%	51%	108%
95% CI	46.61–65.02	39.04–59.21	3.57–5.93	1.20–3.92
UNDER−THROUGH difference (positive mean = UNDER has improved/increased more than THROUGH)				
*DIFFERENCE*				
Mean	7.61	9.13	1.25	0.66
95% CI	− 7.72–22.94	− 8.26–26.51	− 0.71–3.21	− 1.46–2.79
*THROUGH vs. UNDER*				
P-value*	*P* = 0.313	*P* = 0.286	*P* = 0.197	*P* = 0.524
df t-value	20 − 1.036	19 − 1.099	20 − 1.333	22 − 0.647
*ALL (n = 26)*				
Mean	*52.88*	*45.62*	*4.27*	*2.31*
SD	18.47	20.68	2.39	2.60
CV	35%	45%	56%	113%
95% CI	45.79–59.98	37.67–53.56	3.35–5.19	1.31–3.31

*2-sample t-test assuming unequal variances was used. The null hypothesis is that group means are equal, which is rejected when p-value is below significance level of 0.05

*0 years* before operation except for CC-dist where 2 weeks after operation, *2 years* 2 year after operation, *95% CI* 95% confidence interval, *CC dist.* coraco-clavicular distance (mm), *Constant* Constant Shoulder score, *CV* SD/mean (coefficient of variation), *df* degrees of freedom, *Nottingham* Nottingham Clavicle score, *SD* standard deviation, *SST* Simple Shoulder Test score

At baseline, the mean (SD) Nottingham Clavicle Score was 48.8 (15.8), which increased to 97.7 (4.4) at the 2-year assessment in the THROUGH group and from 38.4 (10.3) to 94.3 (17.9) in the UNDER group (Table 1). The Nottingham Clavicular score, Constant score, Simple Shoulder Test score and coracoclavicular distance at baseline and two years after the operation in the THROUGH and UNDER groups. Ligament reconstruction was successful in both groups, but there were no statistically significant differences in the different efficacy scores or in the changes between the groups. There was a modest increase in the coracoclavicular distance in both groups, but the differences between the groups were not statistically significant. For descriptive and inferential statistics, see Tables 1 and 2.

**Fig. 4:**
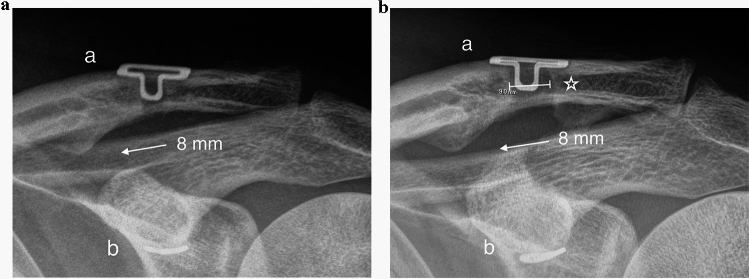
**a** Left shoulder. Postoperative anteroposterior radiograph 2 weeks after surgery. Clavicular clip (**a**) and subcoracoid clip (**b**). The coracoclavicular distance is 8 mm (arrow). In this case, the tendon graft was taken through the coracoid. The THROUGH and UNDER groups cannot be radiologically distinguished. **b** The same left shoulder. Anteroposterior radiograph 2 years after surgery. Clavicular clip (**a**) and subcoracoid clip (**b**). The coracoclavicular distance remained at 8 mm (arrow). The radiological clavicular drill hole diameter increased from the original 6 to 9 mm (star)

The changes were statistically significant within both groups (*P* < 0.001), but the difference between the groups was not statistically significant (Tables 1, 2). Only two patients (one in each group) scored below 90 on the Nottingham Clavicle Score at the 2-year mark. The mean (95% CI) difference in the improvement of the Nottingham Clavicle Score (THROUGH−UNDER) was 7.6 (− 7.2–22.9) units. Similar results demonstrating the effectiveness of the treatment were observed with the Constant and Simple Shoulder Test scores. In the combined dataset, the mean increases from baseline to the 2-year assessment (95% CIs) for the Nottingham Clavicle score, the Constant score, and the Simple Shoulder Test score were 52.9 (45.8–60.0), 45.6 (37.7–53.6), and 4.3 (3.4–5.2), respectively (Table 2). The mean (SD) coracoclavicular distance increased by 1.9 (2.4) and 2.6 (2.8) mm in the THROUGH and UNDER groups, respectively, with no significant difference observed between groups (Table 2). Similarly, the mean (SD) clavicular drill hole diameter at 2 years was 8.10 (0.74) mm and 7.94 (0.77) mm in the THROUGH and UNDER groups, respectively, with no significant difference between groups (Table 1 and Fig. 4).

No significant differences in patient satisfaction were observed between groups. The 95% CI for the mean difference ranged from − 0.11 to 0.74 units on the 0–3 scale (Table 3). In the combined dataset, 23 patients (88.5%) reported an excellent outcome, two (7.7%) reported a good outcome, and one patient (3.8%) in the UNDER group reported a poor outcome. Notably, the clinical scores of the patient with poor patient satisfaction also indicated an unfavorable treatment outcome (Table 1 and Fig. 4).

**Table 3:** Summary of patient satisfaction results

	Satisfaction after 2 years (3 = excellent, 2 = good, 1 = fair, 0 = poor)
*THROUGH*	
Mean	3.00
Median	3
SD	0.00
CV	0%
95% CI	–
*UNDER*	
Mean	2.69
Median	3
SD	0.79
CV	30%
95% CI	2.23–3.00*
*ALL*	
Mean	2.81
Median	3
SD	0.63
CV	23%
95% CI	2.54–3.00*

*Bootstrap non linear method used for confidence interval

## Discussion

The aim of this prospective study was to evaluate whether taking a semitendinosus tendon graft through a 4.5-mm coracoid drill hole would increase the risk of fracture. Clavicular wound healing, shoulder scores, radiological changes and patient satisfaction were also evaluated. In this study, no coracoid fractures occurred. The clavicular wounds healed well, there were no infections, and no protruding objects were detected on the clavicle. There were no statistically significant differences observed in the efficacy scores or their changes between the THROUGH and UNDER groups. Both groups exhibited a significant increase in the Nottingham Clavicle score, Constant score, and Simple Shoulder Test score. The 95% CIs for the improvements observed in the various clinical scores clearly indicated the efficacy of the techniques, and patient satisfaction was also notably high. The mean (SD) coracoclavicular distances increased by 1.9 (2.4) and 2.6 (2.8) mm in the THROUGH and UNDER groups, respectively, which was considered moderate. The mean (SD) clavicular drill hole diameters at 2 years were 8.10 (0.74) mm and 7.94 (0.77) mm in the THROUGH and UNDER groups, respectively.

Many modern techniques include CC ligament reconstruction using a hamstring tendon graft in order to achieve an anatomic stable solution and enhance the longevity of the reconstruction [47, 48]. Several studies have shown that techniques that reconstruct the CC ligament using a tendon graft clearly give better results, especially in chronic cases [22–25]. However, the precise moment when the dislocation becomes truly chronic may be hard to determine [24, 25]. Because the reconstruction with mere suture sling never is fully reliable, the authors use the tendon graft in all CC-reconstructions [48]. Similar to the techniques demonstrated in this study, coracoclavicular reconstruction techniques also effectively address associated lateral clavicular fractures [49].

It is known that the AC joint capsule plays an important role in the anteroposterior stability of the clavicle [6–8]. In earlier studies, it has been shown that reconstructing also the AC joint capsule is needed in order to stabilize the distal clavicle [27–34]. In this study both the CC and AC ligaments were reconstructed with a semitendinosus graft in all cases.

It has earlier been stated that clavicular and coracoid drill-holes housing the tendon graft risk a fracture [36–39]. With the coracoid process that is especially true coracoid being a relatively small bone. In some techniques, the drill holes through the coracoid have been quite large clearly risking a fracture [39]. With smaller correctly positioned coracoid drill holes, mores successful results may be achieved. In an earlier study where the tendon graft was taken through a 4.5-mm drill hole, the occurrence of coracoid fracture was 3.4%. [22] In theory, guiding the tendon graft through both clavicular and coracoid drill holes might create a stronger connection between the clavicle and coracoid since there is a bony channel in both ends to help the graft to firmly heal.

Taking the Semitendinosus graft through the 5.5-mm clavicular drill hole is very practical, especially when the graft limb is extended over the AC joint. The expansion of the clavicular drill holes in this study was moderate, but the phenomenon remains a problem. The largest expansion of the clavicular drill hole was 9 mm. In this technique, the clavicular drill hole houses the 5 × 2-mm Clavicular Clip loop, interconnecting suture and tendon graft. The Clavicular Clip loop goes approximately halfway through the drill hole and it was hoped that its rigidity could prevent excess tunnel widening by reducing the windshield wiper effect [38]. It was also hoped that a Clavicular Clip length of 16–20 mm would eliminate the adverse effects of tunnel widening and decrease pressure on the clavicular cortex to prevent implant sinking. Interestingly, major changes in the clavicular drill hole seem to take place during the first postoperative year [50]. An advantage of the techniques used in this study is that each bone required only one drill hole [42, 43]. It has been earlier suggested that the clavicular drill hole should be smaller. However, a smaller drill hole would mean a slimmer graft which in turn may make the reconstruction weaker [21]. According to our earlies studies and this study there has been only one distal clavicle fracture after forceful trauma and even that healed conservatively and the CC reconstruction remained intact [50]. However, to some extent tunnel widening may be considered inevitable but in techniques like this, drill holes can hardly be totally avoided [36–38].

Although there was slight radiological increase in the coracoclavicular distance, it did not directly affect the efficacy scores or patient satisfaction. The increase probably was due to the expected failure of the interconnecting suture at some point and the tensile tendon graft taking the load [42]. There were also some cases where the distal clavicle remained in an elevated position from the beginning due to not so perfect distal clavicle repositioning. However, these factors did not seem to directly affect the patient satisfaction, given that the distal clavicle healed firmly in its place and was pain free. This was probably due to the tendon graft and reconstruction of both the CC and AC ligaments. A sound surgical technique is not the sole consideration for these patients; it is important to closely follow the postoperative treatment protocol, and the tendon graft must be sufficiently well integrated to the bone channels and surroundings before rehabilitation begins at 8 weeks after surgery. Successful operative treatment of AC dislocations is not easy, but based on our clinical experience, previous studies, and the current study, the results can be favorable.

## Limitations

Numerous different outcome scores have been used in previous studies, and therefore, these results cannot be directly compared with those of other studies. The original aim of recruiting 40 patients in a two-year timeframe was already a formidable task; thus, power analysis was not conducted. The small sample size of 26 cases is also duly acknowledged. The COVID-19 outbreak severely hampered recruitment due to hospital closures and restrictions.

## Conclusions

There were no significant differences between the two groups. There were no implant related complications in the clavicular wound healing. The results support the notion that good results are achieved by reconstructing both the CC and AC ligaments with a tendon graft.

## Data Availability

The datasets generated and analyzed during the current study are available from the corresponding author on reasonable request.
